# Probenecid as a sensitizer of bisphosphonate-mediated effects in breast cancer cells

**DOI:** 10.1186/1476-4598-13-265

**Published:** 2014-12-11

**Authors:** Regina Ebert, Jutta Meissner-Weigl, Sabine Zeck, Jorma Määttä, Seppo Auriola, Sofia Coimbra de Sousa, Birgit Mentrup, Stephanie Graser, Tilman D Rachner, Lorenz C Hofbauer, Franz Jakob

**Affiliations:** Orthopedic Center for Musculoskeletal Research, University of Würzburg, Brettreichstrasse 11, 97074 Würzburg, Germany; Division of Endocrinology, Diabetes and Bone Metabolism, Technical University of Dresden, Fetscherstrasse 74, 01307 Dresden, Germany; School of Pharmacy, University of Eastern Finland, Yliopistonranta 1C, POB 1627, 70211 Kuopio, Finland

**Keywords:** Bisphosphonates, Caspase 3/7 activity, Cell viability, Probenecid, Novobiocin, Breast cancer cells

## Abstract

**Background:**

Anti-resorptive bisphosphonates (BP) are used for the treatment of osteoporosis and bone metastases. Clinical studies indicated a benefit in survival and tumor relapse in subpopulations of breast cancer patients receiving zoledronic acid, thus stimulating the debate about its anti-tumor activity. Amino-bisphosphonates in nM concentrations inhibit farnesyl pyrophosphate synthase leading to accumulation of isopentenyl pyrophosphate (IPP) and the ATP/pyrophosphate adduct ApppI, which induces apoptosis in osteoclasts. For anti-tumor effects μM concentrations are needed and a sensitizer for bisphosphonate effects would be beneficial in clinical anti-tumor applications. We hypothesized that enhancing intracellular pyrophosphate accumulation via inhibition of probenecid-sensitive channels and transporters would sensitize tumor cells for bisphosphonates anti-tumor efficacy.

**Method:**

MDA-MB-231, T47D and MCF-7 breast cancer cells were treated with BP (zoledronic acid, risedronate, ibandronate, alendronate) and the pyrophosphate channel inhibitors probenecid and novobiocin. We determined cell viability and caspase 3/7 activity (apoptosis), accumulation of IPP and ApppI, expression of ANKH, PANX1, ABCC1, SLC22A11, and the zoledronic acid target gene and tumor-suppressor KLF2.

**Results:**

Treatment of MDA-MB-231 with BP induced caspase 3/7 activity, with zoledronic acid being the most effective. In MCF-7 and T47D either BP markedly suppressed cell viability with only minor effects on apoptosis. Co-treatment with probenecid enhanced BP effects on cell viability, IPP/ApppI accumulation as measurable in MCF-7 and T47D cells, caspase 3/7 activity and target gene expression. Novobiocin co-treatment of MDA-MB-231 yielded identical results on viability and apoptosis compared to probenecid, rendering SLC22A family members as candidate modulators of BP effects, whereas no such evidence was found for ANKH, ABCC1 and PANX1.

**Conclusions:**

In summary, we demonstrate effects of various bisphosphonates on caspase 3/7 activity, cell viability and expression of tumor suppressor genes in breast cancer cells. Blocking probenecid and novobiocin-sensitive channels and transporters enhances BP anti-tumor effects and renders SLC22A family members as good candidates as BP modulators. Further studies will have to unravel if treatment with such BP-sensitizers translates into preclinical and clinical efficacy.

**Electronic supplementary material:**

The online version of this article (doi:10.1186/1476-4598-13-265) contains supplementary material, which is available to authorized users.

## Background

Bone metastases occur exceptionally often in cancers derived from the breast, the prostate and the kidney. The biological process of metastasis requires the capability of extravasation, migration and homing to the bone microenvironment. Tumor cells when metastasized to bone are able to activate osteoclasts by secreting osteoclast promoting factors [1]. The latter is the basis of the classical concept of osteolytic metastasis, while the local secretion and/or activation of latent growth factors contribute to the development of osteoblastic metastases and autocrine tumor propagation [2].

Anti-resorptive bisphosphonates (BP) accumulate in bone as they show a high affinity to hydroxylapatite and are incorporated by osteoclasts via phagocytosis [3]. First generation BP like clodronate induce apoptosis by accumulating toxic ATP adducts whereas second generation amino-BP inhibit the mevalonate pathway enzyme farnesyl pyrophosphate synthase (FPPS) very specifically. As a consequence protein prenylation of small GTP binding proteins like Rab, Ras or lamins, which are important for cytoskeleton organization and cellular polarization, is inhibited and may initiate apoptosis [4]. Additionally it was reported for zoledronic acid (ZA) and to a lesser extend for ibandronate (IBN) and risedronate (RIS) as well as for alendronate (ALN) that treatment of cells led to the accumulation of isopentenyl pyrophosphate (IPP) and produced a new endogenous ATP analogue (triphosphoric acid 1-adenosin-5′-yl ester 3-(3-methylbut-3-enyl) ester (ApppI)), which also caused apoptosis in osteoclasts by inhibiting the mitochondrial ADP/ATP translocase [5].

BP have been developed for osteoporosis treatment where numerous clinical studies proved their efficacy in reducing the incidence of fragility fractures. When applied in higher cumulative doses than used for osteoporosis, BP effectively reduced the number of skeletal related events in patients with bone metastases [6, 7], which has made them an important class of drugs in the treatment of osteolytic bone diseases [8]. Besides the effects on their classical targets, cells of the myelomonocytic/macrophage lineage and especially osteoclasts, BP have been shown to induce apoptosis in a variety of benign and malignant cells, although in some cases μM concentrations were required [3]. These *in vitro* effects in concert with clinical studies have stimulated discussions about a putative clinically relevant anti-tumor effect of BP. Almost twenty years ago it was shown that adjuvant treatment with BP reduces the incidence of bone metastases and the overall mortality in patients suffering from breast cancer. These results were confirmed in the ABCSG-12 trial, where ZA was used only twice a year for the adjuvant treatment of estrogen receptor positive breast cancer patients. Positive long term effects from patients of the first cohort were reported in a second analysis more than ten years after the first publication [9–11]. Moreover, a synergistic anticancer efficacy of ZA in combination with neoadjuvant chemotherapy was shown in breast cancer patients with respect to additional tumor shrinkage [12]. These effects were confirmed by the ZO-FAST study, where ZA was associated with improved disease-free survival in postmenopausal women [13]. However, the discussion is ongoing and presently a proven anti-tumor effect seems to be restricted to the postmenopausal high bone turnover subpopulation of women suffering from breast cancer [14].

The detailed characterization of the molecular effects of modern BP like ZA stimulated research about their effects on both osteoblastic differentiation and on anti-tumor effects, but a prominent question remained to be solved, if local μM concentrations of BP can be achieved in the clinical setting [15, 16]. Such high concentrations are needed because the cellular uptake is relatively poor in cells other than macrophages and osteoclasts as described for e.g. free ZA in ovarian tumor cells [17]. However it was speculated that BP concentrations in the bone microenvironment and especially in the resorption lacuna can reach concentrations up to hundreds of μM [18]. The two most prominent *in vitro* effects of BP, which may add to their putative anti-tumor effects, are the capability of inducing apoptosis in tumor cells and eliciting an immune response. Stimulation of breast cancer cells with bisphosphonates and inhibition of the mevalonate pathway as a consequence leads to the accumulation of IPP and ApppI. IPP acts as phosphoantigen for γδT cells, which have the ability to attack the tumor cells [19]. The mechanism by which IPP is secreted or transported to the outer surface of a cell is still unknown [20, 21]. Channels and transporters for pyrophposphates or ATP might be responsible for mediating these effects and promising candidates are pannexin (PANX) hemichannels (especially PANX1), the progressive ankylosis protein homolog ANKH as well as organic anion transporters of the solute carrier family 22 (organic anion transporter SLC22A6, SLC22A8 and SLC22A11) and multidrug resistance associated protein 1 (ABCC1). For PANX1, which is a part of the purinergic receptor P2RX7 complex, participation in ATP release was shown [22–24]. ANKH is a transmembrane protein and controls intra- and extracellular levels of pyrophosphate, which is important in bone mineralization [25]. Solute carrier family 22 members are responsible for the transport of organic anions mainly in the kidney and liver [26] whereas ABCC1, a member of the human ABC transporter family that is involved in multidrug resistance, mediates export of organic anions and drugs from the cytoplasm [27]. All channels and transporters are sensitive to the anion transport blocker probenecid (Prob), whereas carbenoxolone (CBX) has no effect on ANKH but is effective in inhibiting PANX1 mediated release. Ibrutinib was described to block ABCC1 transport while novobiocin inhibits SLC22A6, 8 and 11 [24, 28–31]. Therefore these substances can be used to distinguish between ANKH, PANX1, ABCC1 and SLC22A mediated effects.

Sustained effects of bisphosphonates on osteogenic differentiation upon treatment with low concentrations and intermittent treatment with high concentrations of ZA and alendronate were previously demonstrated [32, 33], while permanent exposure to high doses induced apoptosis in both tumor cells and osteogenic precursors [32, 34, 35]. In MCF-7 cells we identified ZA target genes as KLF2, KLF6 and Ki-67 and we assumed that IPP/ApppI accumulation might mediate this effect in cell populations that are largely insensitive to apoptosis induction [15]. It is of major importance to unravel the differential potency of various BP on tumor cell growth and apoptosis and to describe the downstream targets in non-osteoclastic cells.

Here we show that breast cancer cell lines permanently exposed to various BP (zoledronic acid, ibandronate, alendronate, risedronate) undergo apoptosis (MDA-MB-231, to a lesser extend T47D) or show reduced viability (MCF-7). The relative potency of various BP mirrors their antiosteolytic potency with ZA inducing the greatest increase in apoptosis. Interestingly, all other BP tested were almost equally potent in reducing MCF-7 viability. Co-incubation with the anion transporter and channel blocking agent probenecid and novobiocin revealed a synergistic effect, which shows that accumulated pyrophosphates might be secreted to the extracellular space and according to previously described sensitivity renders SLC22A family members as good candidates for the sensitizing effects. Bisphosphonates have relevant effects on tumor cell biology and an adjuvant therapy with BP in combination with a respective sensitizer might be useful in the treatment of breast cancer.

## Results

### Permanent incubation of breast cancer cell lines with different bisphosphonates modulates cell viability and caspase 3/7 activity

**Figure 1:**
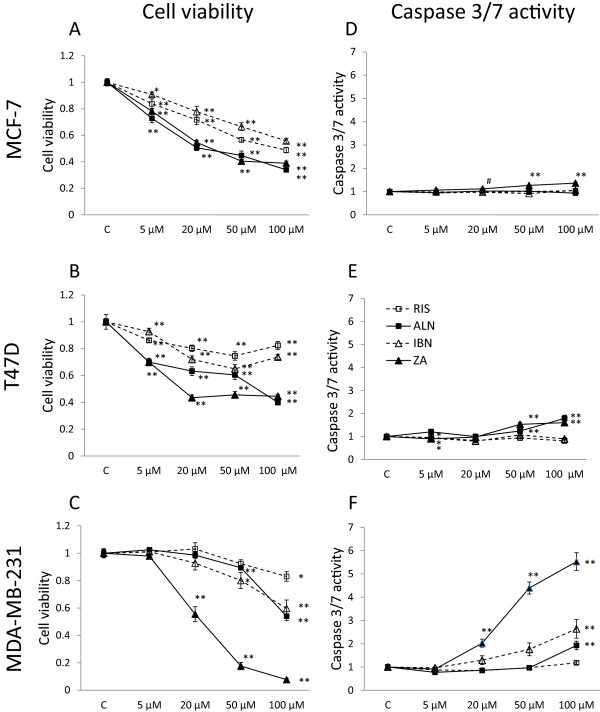
**Cell viability and caspase 3/7 activity in breast cancer cells treated with various bisphosphonates.** Cell viability **(A-C)** and caspase 3/7 activity **(D-F)** in MCF-7, T47D and MDA-MB-231 breast cancer cells treated with 5–100 μM zoledronic acid (ZA, filled triangles), ibandronate (IBN, open triangles), alendronate (ALN, filled squares) and risedronate (RIS, open squares). All data are expressed as means of six different measure points of three independent experiments as percent of controls ± SEM. Significances were calculated with the Mann–Whitney *U* test (**p < 0.001, *p < 0.01, ^#^p < 0.05).

MCF-7, T47D and MDA-MB-231 cells were subjected to various concentrations of ZA, IBN, ALN and RIS (5, 20, 50 and 100 μM) for 72 h (Figure 1). In MCF-7 cell viability was inhibited by all used bisphosphonates (Figure 1A). 100 μM ZA and ALN suppressed the viability to 40%, RIS and IBN to 50 – 60%. In T47D cells ZA inhibited the viability to 40% starting from 20 μM with no increasing effects when higher doses were used. ALN was less potent when applied at 20 and 50 μM but showed the same inhibition at 100 μM. RIS and IBN reduced cell viability only to approx. 70 and 80% in a U-shaped manner when applied in doses of 50 μM and higher (Figure 1B). ZA was most potent in inhibiting the viability of MDA-MB-231 cells (Figure 1C, filled triangles). 20 and 50 μM ZA reduced cell viability to 50 and 20%, respectively. IBN (open triangles) and ALN (filled squares) were less potent, while RIS (open squares) had almost no effect.

In MCF-7 cells only ZA showed marginal effects on caspase 3/7 induction (Figure 1D) while in T47D cells only ZA and ALN slightly enhanced caspase 3/7 activity when applied in 50 and 100 μM doses (Figure 1E). When analyzing caspase 3/7 activity of MDA-MB-231 cells (Figure 1F) treated with different bisphosphonates 100 μM ZA induced a 5-fold enhancement (filled triangles), while IBN (open triangles) was able to increase caspase 3/7 activity 2-fold compared to ALN (filled squares, 1.5-fold) at the same concentration. RIS (open squares) had no effect on caspase 3/7 activity in MDA-MB-231 cells. No effect of ZA on cytotoxicity could be observed (data not shown).

Significances were calculated with the Mann–Whitney *U* test by comparison of the untreated controls to the stimulated values (**p < 0.001, *p < 0.01, ^#^p < 0.05).

### Bisphosphonate treatment induces IPP/ApppI production in breast cancer cells

**Figure 2:**
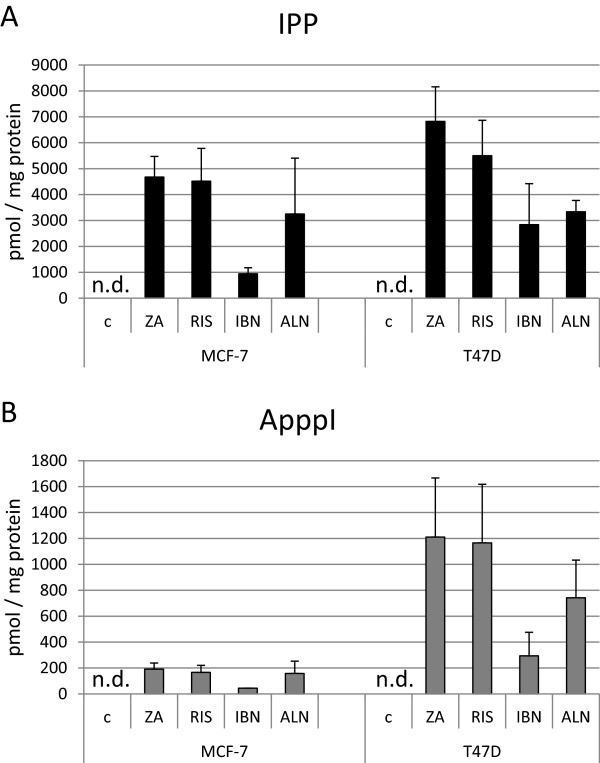
**Detection of IPP and ApppI in breast cancer cells treated with various bisphosphonates.** IPP **(A)** and ApppI **(B)** were measured in bisphosphonate-stimulated MCF-7 and T47D breast cancer cells. All data are expressed as means of three independent experiments ± SEM (ZA: zoledronic acid, RIS: risedronate, IBN: ibandronate, ALN: alendronate, n.d.: not detectable).

The accumulation of IPP and ApppI was analyzed in MCF-7, T47D and MDA-MB-231 breast cancer cells after treatment with the bisphosphonates ZA, RIS, IBN and ALN, respectively. By comparing the three different cell lines high concentrations of IPP were detected in T47D and MCF-7 cells while ApppI concentrations were high in T47D and moderate in MCF-7 cells. No reproducible amounts of IPP and ApppI could be measured in MDA-MB-231 cells as it was reported before [19] (data not shown). In T47D cells ZA induced high amounts of IPP (6,820 pmol/mg protein) while RIS treatment resulted in the accumulation of moderate levels (5,500 pmol/mg protein) (Figure 2A, right bars) in contrast to ALN and IBN, which induced lower IPP accumulation (3,336 pmol/mg protein and 2,838 pmol/mg protein, respectively) although with high variability when IBN was applied. Determination of ApppI revealed similar concentrations after treatment with ZA and RIS (1,210 and 1,165 pmol/mg protein) (Figure 2B, right bars). Determination of ApppI concentrations after ALN treatment showed a moderate induction of 742 pmol/mg protein while IBN treated cells accumulated only 294 pmol ApppI/mg protein. In MCF-7 cells ZA and RIS stimulation resulted in the accumulation of 4,674 and 4,520 pmol IPP/mg protein while values for ALN treated cells were moderate (3,250 pmol/mg protein) with IPP only detectable in two out of three ALN treated samples. IPP concentrations for IBN treated cells were lowest (940 pmol/mg protein) (Figure 2A, left bars). ApppI values in MCF-7 cells were much lower compared to the values observed in T47D cells. In ZA, RIS and ALN stimulated cells ApppI values were between 191 and 156 pmol/mg protein, with ApppI only detectable in two out of three ALN treated samples. ApppI was only measureable in one out of three samples in IBN treated cells (Figure 2B, left bars). In MDA-MB-231 cells IPP and ApppI were detectable in only one out of three samples (data not shown).

### Probenecid co-treatment enhances bisphosphonate effects on cell viability and caspase 3/7 activity

**Figure 3:**
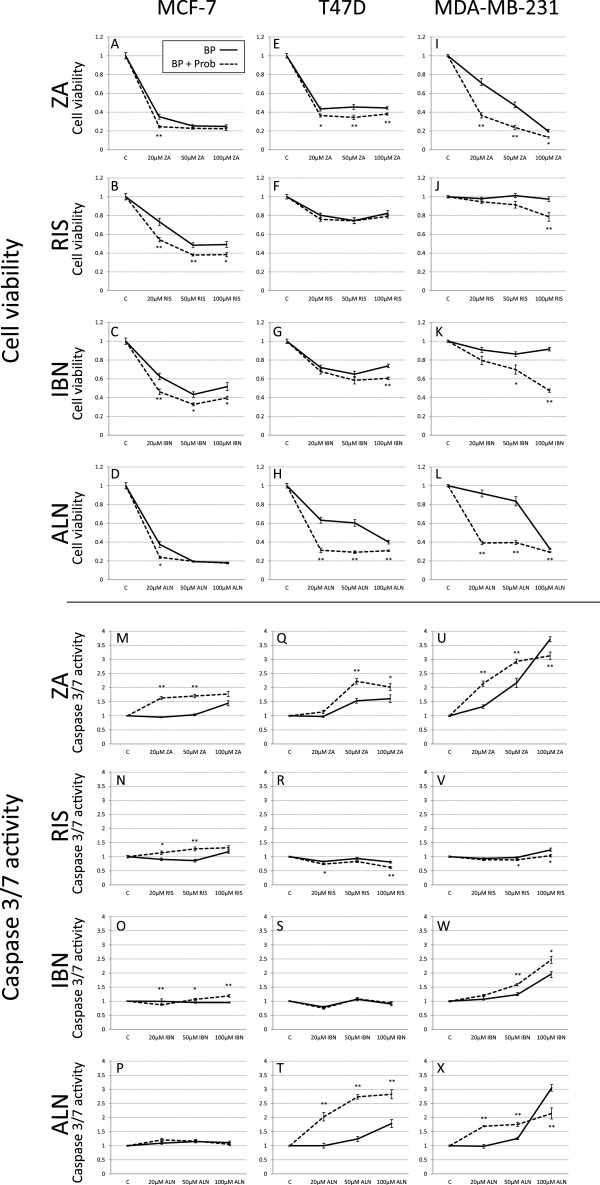
**Cell viability and caspase 3/7 activity in breast cancer cells co-treated with probenecid and bisphosphonates.** Cell viability **(A-L)** and caspase 3/7 activity **(M-X)** was determined in MCF-7, T47D and MDA-MB-231 breast cancer cells after treatment with ZA (zoledronic acid), RIS (risedronate), IBN (ibandronate), ALN (alendronate) alone and in combination with probenecid. All data are expressed as means of six different measure points of three independent experiments as percent of controls ± SEM. Significances were calculated with the Mann Whitney *U* test (**p < 0.005, *p < 0.05). BP: bisphosphonate, black line; Prob: probenecid, dotted line probenecid co-treatment.

MCF-7, T47D and MDA-MB-231 breast cancer cells were stimulated with 20, 50 and 100 μM ZA, RIS, IBN, or ALN, respectively (Figure 3, black lines) and co-treated with 0.25 mM probenecid (Prob, Figure 3, dotted lines) for 72 h. Determination of cell viability in MCF-7 cells (Figure 3A-D) revealed a synergistic effect of probenecid on BP effects compared to BP alone with almost parallel curves in terms of RIS and IBN. In ZA and ALN treated cells, probenecid showed additive effects when submaximal BP doses of 20 μM were applied. With a higher BP dosing the curves almost converged. In T47D cells (Figure 3E-H) Prob and RIS co-stimulation had no additive effect on the inhibition of cell viability compared to cells treated with RIS alone in contrast to ZA or IBN stimulated cells, where Prob co-treatments increased the inhibitory effect of the respective BP. The effects of ZA and IBN obtained in T47D and MCF-7 cells were comparable in contrast to ALN stimulated T47D cells where the pattern of cell viability was different to all other BP. Prob co-stimulation had maximal effects at an ALN range between 20 and 50 μM and depicted much less impact on cell viability at higher BP concentrations. In MDA-MB-231 cells (Figure 3I-L) ZA/Prob and ALN/Prob co-treatment experiments revealed comparable results as well as in RIS/Prob and IBN/Prob treated specimens, respectively. The graphs of RIS and IBN single treated cells diverged from the RIS/Prob and IBN/Prob co-stimulations with a maximum at 100 μM BP, whereas the graphs of ZA and ALN single treated cells diverged from the ZA/Prob and ALN/Prob co-treatments maximally at a concentration of 20 μM BP and converged at higher doses at 100 μM.

Determination of caspase 3/7 activity in BP/Prob co-stimulated MCF-7 cells (Figure 3M-P) revealed an activity induction at concentrations of 20 and 50 μM ZA and RIS after Prob stimulation, whereas the combination of 20 μM IBN and Prob inhibited caspase 3/7 activity in contrast to doses of 50 and 100 μM IBN where Prob had a slight but measurable additive effect. No additive effect of Prob and ALN could be observed. In T47D cells (Figure 3Q-T) no caspase 3/7 activity was induced by RIS and IBN, as we have already shown in Figure 1, and IBN/Prob or RIS/Prob co-stimulations did not show any activity induction, RIS/Prob even reduced the measured caspase 3/7 activity. An additive effect of ZA/Prob was seen compared to ZA single stimulated samples at 50 and 100 μM ZA whereas the combination of ALN and Prob showed massive effects on caspase 3/7 activity induction at all ALN concentrations compared to ALN stimulations alone. When we determined the activity of caspase 3/7 in MDA-MB-231 (Figure 3U-X) after stimulating cells with BP alone or in combination with Prob we observed an additive effect of Prob/BP in combination compared to BP alone in ZA and ALN treated cells at 20 and 50 μM BP, although at higher doses of 100 μM caspase 3/7 activity was diminished in the BP/Prob samples compared to the BP stimulated specimens. IBN/Prob co-treatment increased caspase 3/7 activity compared to IBN single stimulated cells at all concentrations whereas in RIS treated cells RIS/Prob co-treatment had the opposite effect and caspase 3/7 activity was reduced.

Significances were calculated with the Mann–Whitney *U* test by comparison of the BP stimulated samples to the BP/Prob co-treated values (**p < 0.005, *p < 0.05).

### Probenecid enhances BP-induced IPP/ApppI accumulation

**Figure 4:**
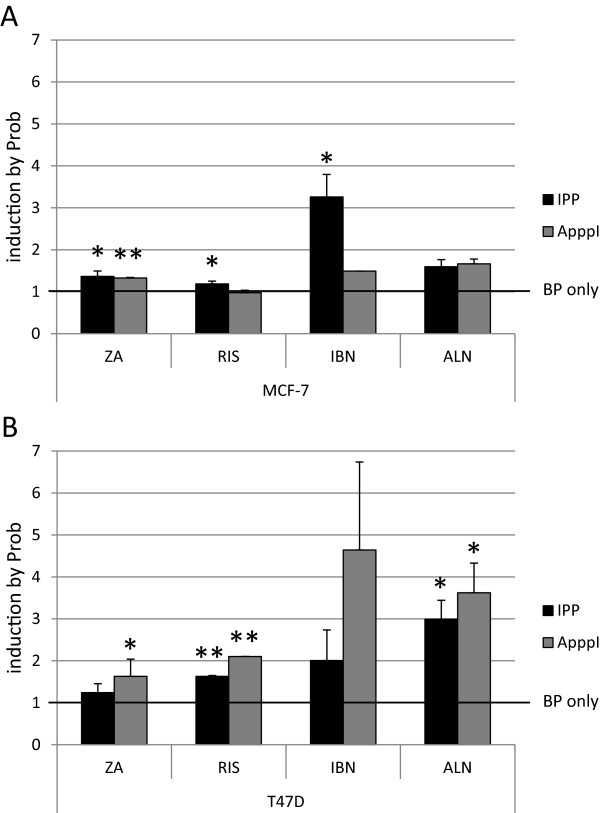
**Induction of IPP and ApppI in bisphosphonate-stimulated breast cancer cells by probenecid.** MCF-7 **(A)** and T47D **(B)** cells were treated with ZA (zoledronic acid), RIS (risedronate), IBN (ibandronate), ALN (alendronate) alone and in combination with probenecid (Prob). All data are expressed as means of three independent experiments ± SEM. The induction of IPP (black bars) and ApppI (grey bars) in BP stimulated cells by Prob is shown. Significances were calculated with the Mann–Whitney *U* test (**p < 0.005, *p < 0.05).

IPP and ApppI accumulation was measured in breast cancer cells after co-stimulation with bisphosphonates and probenecid. In MCF-7 cells (Figure 4A) Prob co-treatment significantly increased the BP induced accumulation of IPP (black bars) in ZA, RIS and IBN treated samples. The highest effect was obtained after IBN/Prob co-stimulation, where a 3.2-fold increase of IPP values was obtained compared to IBN treatment alone. The determination of ApppI revealed only a significant additive effect of Prob on ZA treated samples (grey bars). In only two out of three ALN/Prob co-stimulated samples IPP and ApppI could be detected while only one out of three IBN/Prob samples depicted ApppI accumulation. In T47D cells (Figure 4B) Prob co-treatment increased the BP induced accumulation of IPP (black bars) and ApppI (grey bars) with significant values in RIS and ALN specimens in terms of IPP and significant values in ZA, RIS and ALN treated samples in terms of ApppI accumulation. The combination Prob/ALN was most effective with a 3-fold increase in IPP and a 3.5-fold increase in ApppI accumulation compared to ALN treated samples alone. In MDA-MB-231 cells IPP could be detected after ZA/Prob and ALN/Prob co-treatment. All other samples were negative for IPP and ApppI, respectively (data not shown). Significances were calculated from the means of three independent experiments with the Mann–Whitney *U* test (**p < 0.005, *p < 0.05).

### Probenecid co-treatment enhances bisphosphonate-induced expression of the target gene KLF2

We have previously identified KLF2 as a target gene of ZA in MCF-7 cells [15] and postulated its specific upregulation by intracellular BP effects, e.g. IPP/ApppI accumulation and inhibition of protein prenylation. We analyzed if other BP are also able to modulate KLF2 expression in breast cancer cells and if probenecid can enhance the observed effects. In MCF-7 cells ZA induced a 13-fold increase in KLF2 expression, which was further enhanced by Prob co-treatment (32.4-fold expression) compared to untreated controls (Table 1). Additive effects of Prob were also observed when using ALN. The bisphosphonate alone induced KLF2 expression by the factor 5.8 with a further stimulatory effect of Prob co-treatment to a 36.1-fold induction. IBN alone had no impact on KLF2 expression but with Prob co-stimulation the expression of KLF2 increased 6.1-fold in contrast to RIS, where no co-stimulatory effect of Prob on the absent RIS effect could be observed.

**Table 1:** **Effects of co**-**treatment of breast cancer cell lines with probenecid and bisphosphonates on the expression of KLF2**

KLF2 expression	MCF-7	T47D	MDA-MB-231
ZA 20 μM	13.0** (2.3-60.8)	3.0* (1.2-7.6)	3.1 (0.6-16.0)
ZA + Prob	32.4** (5.8-198.5)	2.6* (1.0-6.7)	5.1* (0.7-25.6)
RIS 50 μM	1.6 (0.3-10.1)	2.1* (1.0-3.7)	3.5* (0.6-18.8)
RIS + Prob	4.2 (0.7-35.9)	1.7 (0.7-4.7)	3.4 (0.5-19.2)
IBN 50 μM	2.4 (0.5-15.2)	2.2* (0.9-4.9)	2.4 (0.3-17.3)
IBN + Prob	6.1* (0.8-31.7)	2.2* (0.9-5.9)	4.8** (0.7-28.4)
ALN 50 μM	5.8* (1.2-33.4)	2.0 (0.8-5.5)	1.4 (0.2-11.4)
ALN + Prob	36.1** (9.7-141.4)	1.8 (0.8-5.6)	3.2 (0.4-31.1)
Prob	1.0 (0.3-5.0)	1.0 (0.8-1.3)	1.3 (0.1-9.4)

The expression ratios of KLF2, in MCF-7, T47D and MDA-MB-231 breast cancer cells after treatment with ZA (zoledronic acid), RIS (risedronate), IBN (ibandronate), ALN (alendronate) alone and in combination with probenecid (Prob) compared to untreated controls and normalized to 36B4 (acidic ribosomal phosphoprotein P0) are shown. (**p < 0.001, *p < 0.01 calculated with REST [38]).

In MDA-MB-231 cells ZA and IBN had no significant impact on KLF2 expression but Prob was able to increase KLF2 expression 5.1-fold in ZA and 4.8-fold in IBN co-stimulatory experiments. RIS alone increased KLF2 expression by the factor 3.5 but Prob co-treatment abundant the effect to a non-significant expression. No effect was seen when ALN was used, independent of Prob co-treatment.

In T47D cells no additive effect of Prob was detectable. ZA increased KLF2 expression 3.0 fold but Prob had no additive effect (2.6-fold expression) just as in terms of IBN, where both IBN and IBN/Prob treated samples showed an upregulation of KLF2 by the factor 2.2. RIS alone increased KLF2 expression by the factor 2.1 but no significant enhancement was detectable in RIS/Prob treated cells. ALN alone or the combination ALN/Prob did not influence the expression of KLF2.

### Breast cancer cells express probenecid sensitive channels and transporters

**Figure 5:**
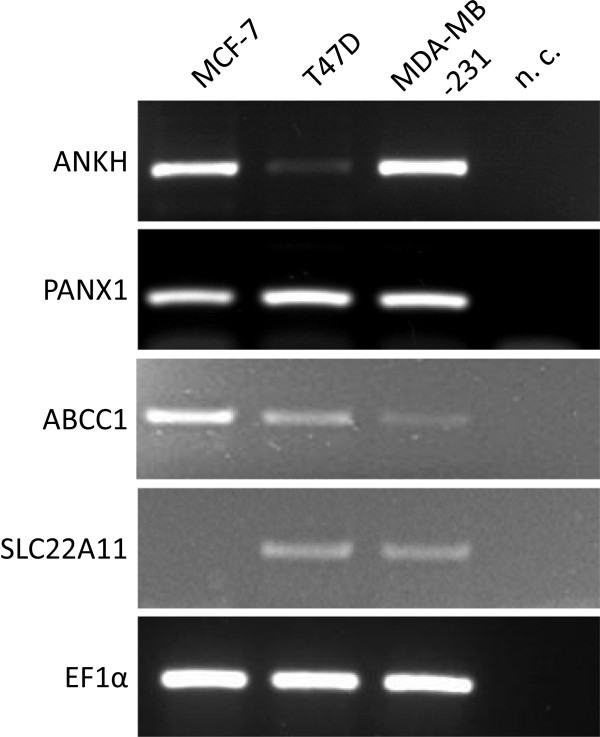
**Expression of probenecid-sensitive channels and transporters in breast cancer cells.** RT-PCR detection of ANKH (progressive ankylosis protein homolog), PANX1 (pannexin 1), multidrug resistance associated protein 1 (ABCC1) and SLC22A11 (solute carrier family 22 member 11) in MCF-7, T47D and MDA-MB-231 cells. EF1α (eukaryotic elongation factor 1 α) was amplified as a housekeeping gene (n.c.: negative control).

The expression of the pyrophosphate channel ankylosis protein homolog (ANKH), the hemichannel protein pannexin 1 (PANX1), multidrug resistance associated protein 1 (ABCC1) and solute carrier family 22 (organic anion transporter) member 6, 8 and 11 (SLC22A6, SLC22A8, SLC22A11) were analyzed in breast cancer cells. PANX1 transcripts could be detected in high amounts in all tested cell lines. ANKH was highly expressed in MCF-7 and MDA-MB-231 cells in contrast to T47D cells where only a faint PCR band was visible. ABCC1 was highly expressed in MCF-7 cells and lower in T47D and MDA-MB-231 cells. SLC22A11 was expressed in T47D and MDA-MB-231 but not in MCF-7 cells (Figure 5). SLC22A6 and SLC22A8 mRNAs were not detectable in all analyzed breast cancer cell lines. QPCR quantification of ANKH expression revealed a 0.18-fold (p < 0.05) lower expression in MCF-7 cells and a 0.07-fold (p < 0.001) lower expression in T47D cells compared to MDA-MB-231 cells whereas PANX1 and ABCC1 expression varied between the cell lines but without any significance. Values were normalized to 36B4 expression (MDA-MB-231 vs. MCF-7) and GAPDH (MDA-MB-231 vs. T47D). Immunocytochemical staining of ANKH and PANX protein confirmed these results with MCF-7 and MDA-MB-231 cells expressing high levels, and T47D expressing low levels of ANKH while PANX1 was equally expressed among the cell lines (Additional file 1: Figure S1).

### ANKH overexpression does not alter probenecid response of BP effects on cell viability

Expression of ANKH in stably transfected T47D cells (T47D-pCMV-ANKH) was confirmed by RT-PCR on mRNA (Additional file 2: Figure S2A) and by immunocytochemistry on protein level (Additional file 2: Figure S2B). When ANKH overexpression T47D cells and T47D control cells carrying the empty pCMV vector were stimulated with 20 and 50 μM ZA (Additional file 2: Figure S2C, black line) and co-stimulated with 0.25 mM Prob (Additional file 2: Figure S2C, dotted line) no difference between the two cell lines was observed in terms of cell viability and caspase 3/7 activity.

### Novobiocin but not carbenoxolone or ibrutinib co-treatment modulates bisphosphonate effects on cell viability and caspase 3/7 activity in MDA-MB-231 breast cancer cells

**Figure 6:**
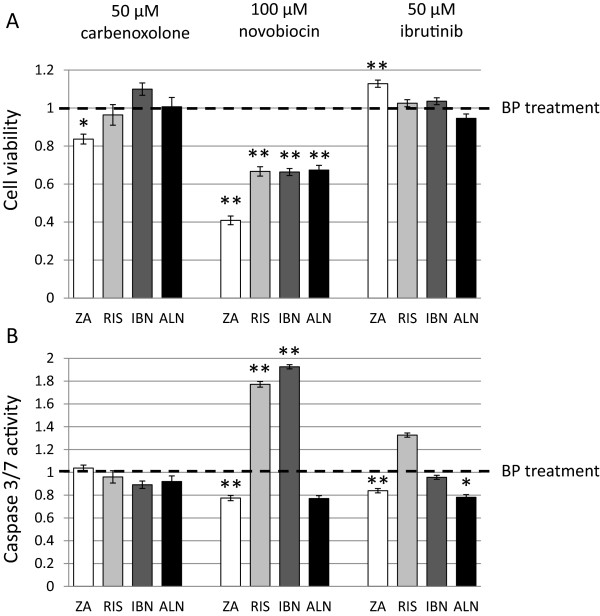
**Cell viability and caspase 3/7 activity in MDA-MB-231 cells co-treated with carbenoxolone, novobiocin, ibrutinib and bisphosphonates.** Cell viability **(A)** and caspase 3/7 activity **(B)** was determined after treatment with ZA (zoledronic acid), RIS (risedronate), IBN (ibandronate), ALN (alendronate) in combination with carbenoxolone, novobiocin and ibrutinib. All data are expressed as means of three different measure points of three independent experiments ± SEM and were normalized to BP treatment alone. Significances were calculated with the Mann Whitney *U* test (*p < 0.05; **p < 0.005).

To further identify the putative channel or transporter responsible for the observed synergistic effects of Prob on BP treatment we applied additional blockers for pyrophosphate channels, organic anion transporters and blockers for multidrug resistance associated protein 1. MDA-MB-231 breast cancer cells were stimulated with 50 μM ZA, RIS, IBN, or ALN, respectively and co-treated with 50 μM carbenoxolone (CBX), a blocker of PANX1, 100 μM novobiocin, a blocker for solute carrier family 22 member 6, 8 and 11 (SLC22A6, SLC22A8, SLC22A11) and 50 μM ibrutinib, an inhibitor for multidrug resistance associated protein 1 (ABCC1) for 72 h. Determination of cell viability showed a synergistic effect on the inhibition of cell viability of CBX and ZA compared to ZA alone in MDA-MB-231 cells, all other combinations had no significant effects (Figure 6A). No synergistic effect of CBX in terms of caspase 3/7 activity induction compared to bisphosphonates stimulations alone could be observed (Figure 6B). Novobiocin plus BP synergistically and highly significantly reduced cell viability of MDA-MB-231 cells with novobiocin/ZA being the most potent combination compared to BP stimulations alone (Figure 6A). Caspase 3/7 activity was synergistically and significantly induced by the combination novobiocin/RIS and novobiocin/IBN while novobiocin/ZA decreased caspase 3/7 activity compared to BP treatment alone (Figure 6B). Ibrutinib plus ZA significantly induced cell viability compared to BP treatment alone (Figure 6A) while caspase 3/7 activity was significantly decreased by the combination ibrutinib/ZA and ibrutinib/ALN compared to BP alone (Figure 6B). Carbenoxolone, novobiocin and ibrutinib alone did not influence cell viability and caspase 3/7 activity (data not shown). Significances were calculated with the Mann–Whitney *U* test by comparison of the BP stimulated samples to the BP/CBX co-treated values (*p < 0.05; **p < 0.005).

## Discussion

Apart from osteoclasts, BP may have clinically relevant effects on benign and malignant cells. We found variable efficacies of different BP on cell viability and caspase 3/7 activity of the breast cancer cell lines MDA-MB-231, T47D and MCF-7. The most potent BP in MDA-MB-231 cells with respect to caspase 3/7 activity induction was ZA, while other BP were markedly less effective in the descending order IBN > ALN > RIS when applied in equimolar concentrations. In the apoptosis insensitive cell lines the picture was different with ZA showing high efficacy on the reduction of cell viability in T47D cells followed by ALN, IBN and RIS in contrast to MCF-7 cells where ZA and ALN depicted comparable effects followed by the weaker compounds RIS and IBN. The observed differences cannot be explained by the rank order of BP in their potency to inhibit the target enzyme farnesyl pyrophosphate synthase (FPPS) with ZA and RIS depicting the highest potency followed by the much weaker inhibitors IBN and ALN [4]. Differences in cellular BP uptake and retention might be responsible for these observations. Nothing is known if all BP are incorporated with the same efficacy, also the mechanism by which tumor cells take up BP is under discussion. The process of pinocytosis might be relevant but the transport through a channel protein cannot be excluded. At pH 7.4 the Amino-BP differ in their zeta potential as the R2 groups of ZA, ALN and IBN are positively charged in contrast to RIS, where the group is negatively charged [4]. Analyses with nanoparticles revealed that positively charged particles are more likely engulfed by pinocytosis than negatively charged particles [36] but also a channel protein or a transporter might distinguish between the different groups in favor of the positively charged BP. Both processes would lead to reduced RIS uptake possibly explaining the weak effects of this compound in tumor cells.

The determination of IPP accumulation and ApppI formation revealed differences between the analyzed breast cancer cell lines and the various BP. In T47D cells we detected high levels of IPP/ApppI and in MCF-7 cells high to moderate levels of IPP and low levels of ApppI as reported previously [19]. In MDA-MB-231 cells IPP and ApppI were only measurable in single samples. ZA was the most potent BP in inducing IPP/ApppI followed by RIS and ALN and IBN being the weakest compound. Our data are not in line with observations in J774 macrophages where ApppI was highest after ZA treatment followed by RIS, IBN and ALN [5], which is similar to their known order of affinity to FPPS and we again speculate that cells incapable of phagocytosis reflect mechanisms for BP uptake, which distinguish between differently charged BP.

Tumor cells are capable of releasing IPP to the extracellular space, which can bind to an unknown antigen-presenting molecule to be recognized by the T-cell receptor of γδT-cells [20, 21]. The mechanisms by which IPP is secreted are unknown and we assumed that the pyrophosphate channels PANX1 and/or ANKH or organic anion transporters as ABCC1 and/or members of the organic anion transporter family SLC22A might mediate this release. All analyzed breast cancer cells depicted similar expression levels of PANX1 and ABCC1 whereas a considerable variability of ANKH and SLC22A11 expression was observed. At first our lead candidate was ANKH but by establishing ANKH transgenic T47D cells we were able to exclude its relevance. We further hypothesized that blocking the above mentioned channels and transporters and subsequently inhibiting the release of BP-induced pyrophosphates enhances IPP/ApppI accumulation, leading to an increase in the BP effect on tumor cell viability. Co-stimulation with the PANX1 inhibitor CBX or the ABCC1 inhibitor ibrutinib together with BP did not result in an appreciable synergistic effect in contrast to a co-stimulation with BP and the organic anion transporter and pyrophosphate channel blocking agent probenecid (Prob) or the SLC22A blocker novobiocin. Both probenecid and novobiocin revealed remarkable additive effects on BP-mediated cell viability reduction and caspase 3/7 activity induction in certain conditions. Therefore we hypothesize that solute carrier family 22 (organic anion transporter) members might be the main candidates to release IPP into the extracellular space. By blocking SLC22A members the described effects of BPs on tumor cells can be intensified.

Furthermore we tried to find out if the additive effect of Prob and BP on tumor cell viability is consistent with an increase in intracellular IPP and ApppI. The most remarkable induction of pyrophosphate accumulation was observed in samples showing low BP-induced IPP/ApppI levels like in IBN and ALN treated T47D cells. T47D cells are generally able to accumulate IPP/ApppI in high amounts as it was reported before [19]. MCF-7 lack the expression of SLC22A11 while T47D show only low expression of ANKH in contrast to MDA-MB-231 cells. MDA cells produce comparably high levels of the three channels/transporters ANKH, PANX1 and SLC22A11 and this is a possible explanation why the intracellular levels of IPP and consecutively ApppI can not be measured.

Equimolar concentrations of IPP and AMP are necessary for the formation of ApppI, catalyzed by aminoacyl-tRNA synthase enzymes. The concentration of AMP is dependent on the cellular energy metabolism. ApppI formation sequestrates AMP, which is then not available for mitochondrial ATP regeneration and ApppI itself blocks the adenine nucleotide translocases, which catalyzes the exchange of cytoplasmic ADP with mitochondrial ATP across the mitochondrial inner membrane. The molecular consequences of ATP deficiency are a negative energy balance and either reduction of proliferation or apoptosis induction, the latter being dependent on the individual susceptibility of cells to induce the apoptosis program. This condition is perfectly reflected by the ATP-based proliferation measurement, which we used for the determination of cell viability. The intracellular pool of nucleotides for energy metabolism and nucleic acid synthesis appears to be different in the used cell lines. In apoptosis sensitive cells this leads to caspase 3/7 activity induction while in resistant cells proliferation is inhibited.

Our data may also shed light on the mechanisms of regulation of intracellular versus extracellular concentrations of phosphate compounds through channel-mediated release in general. As we showed earlier, ZA enhanced mineralization of osteogenic precursors *in vitro*[32]. Inorganic pyrophosphates are inhibitors of mineralization and upon inhibition of the delivery of these pyrophosphates to the cell surface through both stimulation of intracellular decoy mechanisms and inhibition of channel delivery mineralization should be increased around cells that are able to perform coordinated mineralization processes. Further research will have to unravel this putatively pathology-relevant role of channel activity.

## Conclusion

In summary, we report an antitumor activity of all amino-BP, which can be enhanced through inhibition of a putative channel for IPP and by the consecutive rise of intracellular substrates and products of ATP-derived adducts. Probenecid, approved as an uricosuric compound, which inhibits the reabsorption of uric acid, and the antibiotic novobiocin, are accredited compounds. If the effect of enhancing anti-tumor effects of BP using concomitant probenecid or novobiocin treatment can be translated into preclinical and clinical settings without deleterious off-target effects remains to be proven.

## Methods

### Cell culture

All media were obtained from Life Technologies GmbH (Darmstadt, Germany), fetal calf serum (FCS) was obtained from Biochrom AG (Berlin, Germany). MCF-7, MDA-MB-231 and T47D cells were cultivated as described previously [15]. As all experiments were performed with cell lines an ethical approval was not required.

### Establishment of stable ANKH overexpressing T47D cells

2.5 × 10^5^ T47D cells per well were seeded on 6well plates and transfected with 2.5 μg pCMV-ANKH (Sino Biological Inc., Beijing, PR China) or the empty pCMV vector, both linearized with SspI (New England Biolabs, Frankfurt, Germany), by using LipofectAMINE 2000 (Life Technologies GmbH, Darmstadt, Germany) according to the manufacturer’s instructions. As selection antibiotics 100 μg/ml hygromycin (Life Technologies GmbH) was added with every medium change.

### Determination of cell viability and caspase 3/7 activity

For determination of effects of bisphosphonates on cell viability and caspase 3/7 activity MDA-MB-231, T47D and MCF-7 as well as T47D-pCMV-ANKH and T47D-pCMV control cells were seeded on 96-well plates with a density of 1000 cells/well and were stimulated with 5, 20, 50 and 100 μM zoledronic acid (ZA), ibandronate (IBN), alendronate (ALN) and risedronate (RIS) (AXXORA GmbH, Lörrach, Germany) for 72 h. To analyze effects of probenecid (Prob) co-treatment MCF-7, MDA-MB-231 and T47D cells were stimulated with 0.25 mM Prob (Sigma Aldrich GmbH) together with 20, 50 or 100 μM ZA, ALN, RIS and IBN, respectively. Additional co-stimulatory experiments were performed by using 50 μM carbenoxolone (CBX, Sigma Aldrich GmbH), 5 μM ibrutinib, 100 μM novobiocin (both Selleckchem, Houston, USA) together with 50 μM of each bisphosphonate. Cell viability and caspase 3/7 activity were determined after 72 h with the CellTiter-Glo Luminescent Cell Viability Assay and the Caspase-Glo 3/7 Assay (both Promega GmbH, Mannheim, Germany) according to the manufacturer’s instructions as described previously [15]. Cytotoxicity was determined in MCF-7 and MDA-MB-231 cells after ZA treatment by using the CytoTox-Fluor™ Cytotoxicity Assay (Promega GmbH) according to the manufacturer’s instructions. Significances were calculated with the Mann–Whitney U Test by comparison of the untreated control to the stimulated values and by comparison of BP treated cells to BP/Prob or BP/CBX co-stimulated cells.

### RT-PCR

Total RNA was isolated from MCF-7, T47D and MDA-MB-231 cells by using the NucleoSpin RNA II kit (Macherey-Nagel, Düren, Germany) according to the manufacturer’s instructions. Two micrograms of total RNA were reverse-transcribed with MMLV reverse transcriptase (Promega GmbH) in a volume of 25 μl. For amplification of ABCC1, ANKH, PANX1, SLC22A6, SLC22A8, SLC22A11 and the housekeeping gene EF1α 1 μl of cDNA was used as a template in a volume of 50 μl. Taq DNA polymerase was obtained from Promega GmbH and primers were obtained from biomers GmbH, Ulm Germany with the following sequences in 5′-3′ direction: ABCC1_for_ GGATTTTTGCTGTGGATCGT; ABCC1_rev_ ACCAGCCAGAAAGTGAGCAT; ANKH_for_ AAAGCCGTCCTGTGTATGGT; ANKH_rev_ CAGGGATGATGTCGTGAATG; PANX1_for_ AGAGCGAGTCTGGAAACC; PANX1_rev_ CAAGTCTGAGCAAATATGAGG; SLC22A6_for_ GTCTGCAGAAGGAGCTGACC; SLC22A6_rev_ GTCCACAGCACCAAAGATCA; SLC22A8_for_ CTGAGCACCGTCATCTTGAA; SLC22A8_rev_ TGGTGTCCACCAGGATGATA; SLC22A11_for_ CTGCCCTCTTGCTCAGTTTC; SLC22A11_rev_ CACTGGCGTTGGAAAGAGTT; EF1α_for_ AGGTGATTATCCTGAACCATCC; EF1α_rev_ AAAGGTGGATAGTCTGAGAAGC. PCR conditions were as follows: 30 s, 94°C; 30 s, annealing temperature (54°C EF1α, 55°C ANKH, 57°C PANX1, 60°C ABCC1, SLC22A6 and SLC22A11, 62°C SLC22A8), 30 s, 72°C; 35 cycles. PCR bands were analyzed by agarose gel electrophoresis.

### Quantitative PCR

MDA-MB-231, T47D and MCF-7 cells were treated for 72 h with 20 or 50 μM ZA, RIS, IBA, ALN as indicated and co-treated with 0.25 mM probenecid. Quantitative PCR (qPCR) was performed in 20 μl by using 1 μl of the cDNA, which was previously diluted 1:5 and 10 μl of KAPA SYBR FAST qPCR Universal Mix (Peqlab Biotechnologie GmbH, Erlangen, Germany) and 2.5 μl of primer pairs for human *KLF2* or *GAPDH* as housekeeping gene (Quantitect Hs_KLF2_1 and Hs_GAPDH_1_SG, Qiagen GmbH, Hilden, Germany), dissolved according to the manufacturer’s instructions. The primers for 36B4, which was used as housekeeping gene, and the primers for ABCC1, ANKH, and PANX1 (see above) were obtained from biomers.net GmbH, Ulm, Germany and were used in a concentration of 1 pmol each per reaction with the following sequences in 5′-3′ direction: 36B4_qFor: TGCATCAGTACCCCATTCTATCAT; 36B4_qRev: AGGCAGATGGATCAGCCAAGA [37]. QPCR conditions were as follows: 95°C, 3 min; 40 cycles: 95°C, 15 s; 60°C, 15 s; 72°C, 20 s; followed by melting curve analysis for specificity of qPCR products. QPCR was performed with the Opticon DNA Engine (MJ Research, Waltham, USA). Data were obtained from three independent experiments and qPCRs were performed three times. Results were calculated with the Relative Expression Software Tool (REST 2009 V2.0.13) obtained from Qiagen GmbH [38].

### Immunocytochemistry for ANKH and PANX1

Breast cancer cells were seeded on coverslips in 6well plates, grown over night, washed thrice with PBS, fixed for 5 min with ice-cold methanol, dried and stored at −80°C until staining. Before staining cells were washed with PBS, permeabilized with PBS/0.05% Tween-20, washed again with PBS, and blocked with 3% BSA in PBS. Cells were incubated with the primary antibodies for ANKH (1:300 (sc-67242) and PANX1 (1:500 sc-49695), respectively (both Santa Cruz Biotechnology, Inc., Heidelberg, Germany) for 16 h at 4°C and a phycoerythrin-labeled secondary antibody (NorthernLights anti-mouse IgG-NL557, RnD Systems, NL007, 1:400) for 2 h at RT. The coverslips were transferred on slides with a drop of Vectashield with DAPI (LINARIS GmbH, Wertheim, Germany) and analyzed under a fluorescence microscope (Axioskop2, filters 1 and 20, Carl Zeiss MicroImaging GmbH, Jena, Germany).

### Determination of IPP and ApppI in cell samples

IPP and ApppI were determined as described previously [5]. Briefly, 1 × 10^6^ MCF-7, MDA-MB-231 and T47D cells/well were seeded on 6well plates and incubated overnight. MCF-7 cells were stimulated with 20 μM ZA and 50 μM of all other BP (RIS, IBN, ALN), MDA-MB-231 and T47D cells were treated with 50 μM BP (ZA, RIS, IBN, ALN), 0.25 mM Prob or the combination of each BP and Prob for 24 h, washed with ice-cold PBS and harvested. IPP/ApppI was extracted with ice-cold acetonitrile (300 μl) and water (200 μl) containing 0.25 mM NaF and Na_3_VO_4_ as phosphatase inhibitors. IPP and ApppI were quantified with HPLC-ESI-MS, protein contents were determined by the BCA method (Perbio Science Deutschland, Bonn, Germany). Values were obtained from three independent experiments.

## Electronic supplementary material

Additional file 1: Figure S1: Immunocytochemical staining of ANKH and PANX1, nuclei are stained with DAPI. Representative images are shown, the bar represents 100 μm. (PDF 447 KB)

Additional file 2: Figure S2: Overexpression of ANKH in T47D cells. **A)** Amplification of ANKH in pCMV-ANKH and pCMV stable cell lines. EF1α was used as a housekeeping gene. **B)** Immunocytochemical staining of ANKH in pCMV-ANKH and pCMV stable T47D cells. Nuclei are stained with DAPI. Representative images are shown, the bar represents 100 μm. **C)** Cell viability and caspase 3/7 activity in pCMV-ANKH and pCMV stable T47D cells co-treated with probenecid and ZA. All data are expressed as means of six different measure points of three independent experiments as percent of controls ± SEM. BP: bisphosphonate, black line; Prob: probenecid, dotted line probenecid co-treatment. (PDF 344 KB)

## Abbreviations

ABCC1: ATP-Binding Cassette, Sub-Family C (CFTR/MRP), Member 1
ALN: Alendronate
ANKH: progressive ankylosis protein homolog
ApppI: triphosphoric acid 1-adenosin-5-yl ester 3-(3-methylbut-3-enyl) ester
BP: Bisphosphonates
CBX: Carbenoxolone
DAPI: 4′,6-diamidino-2-phenylindole
EMT: Epithelial mesenchymal transition
FCS: Fetal calf serum
FPPS: Farnesyl pyrophosphate synthase
GAPDH: Glyceraldehyde-3-phosphate dehydrogenase
hMSC: human mesenchymal stem cells
IBN: Ibandronate
IPP: Isopentenyl pyrophosphate
PANX: Pannexin
PBS: Phosphate buffered saline
Prob: Probenecid
qPCR: quantitative polymerase chain reaction
RIS: Risedronate
SLC: Solute carrier family
ZA: Zoledronic acid.
