# Supplementary figures and images for: Probenecid as a sensitizer of bisphosphonate-mediated effects in breast cancer cells
Source: Mol Cancer. 2014 Dec 11;13:265. doi: 10.1186/1476-4598-13-265 (PMC4295226; doi:10.1186/1476-4598-13-265)

MCF-7

T47D

MDA-MB-231

ANKH

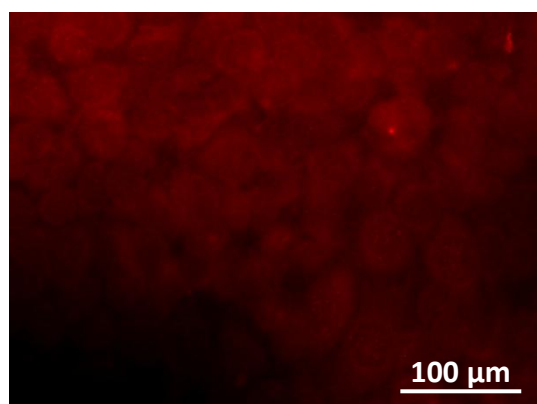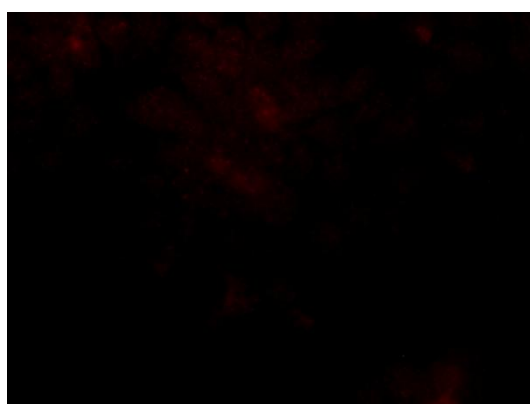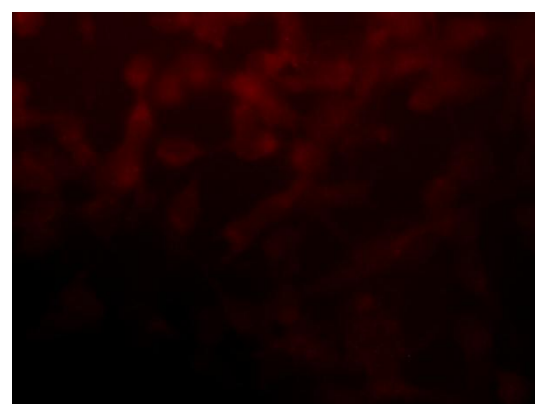

DAPI

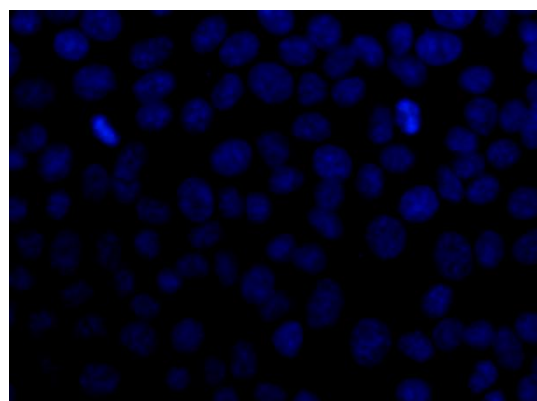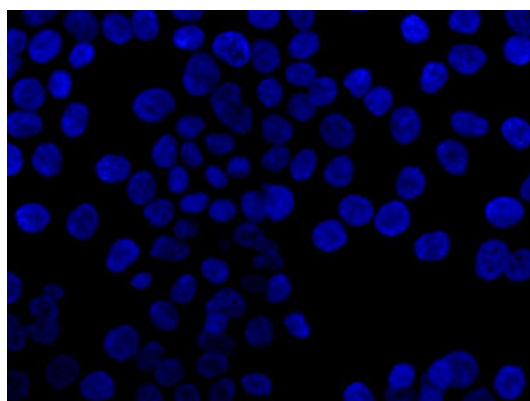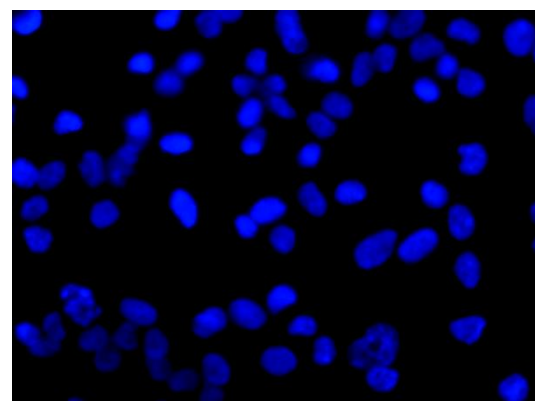

merge

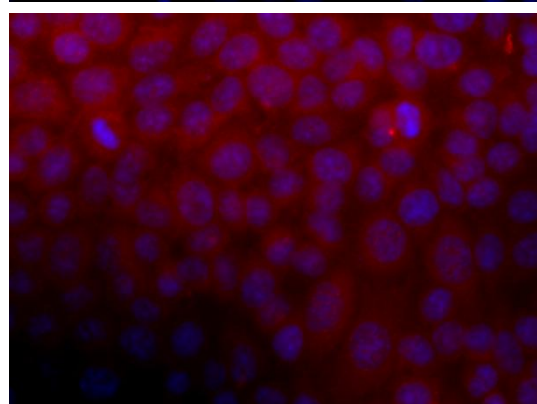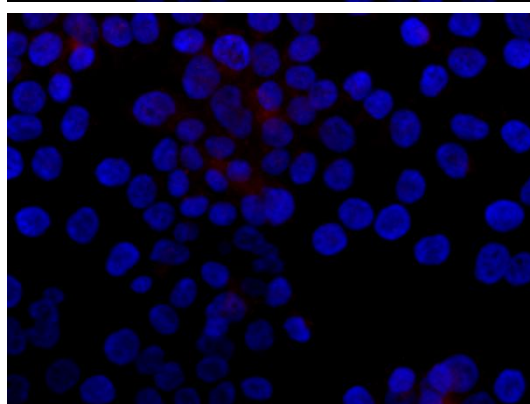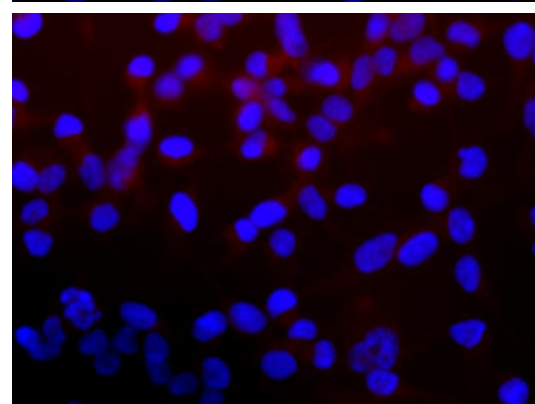

PANX1

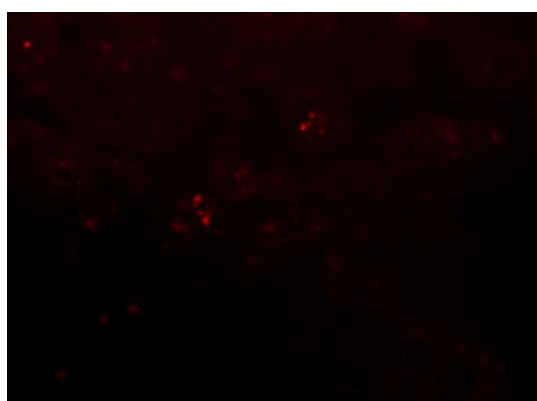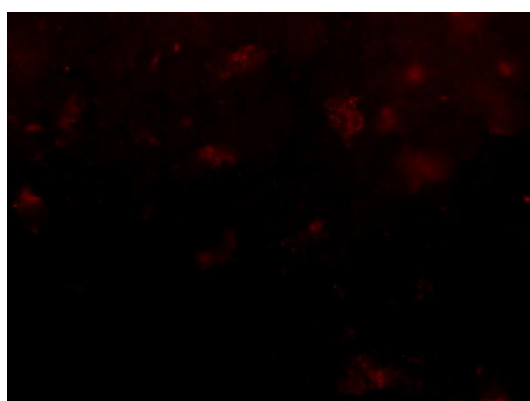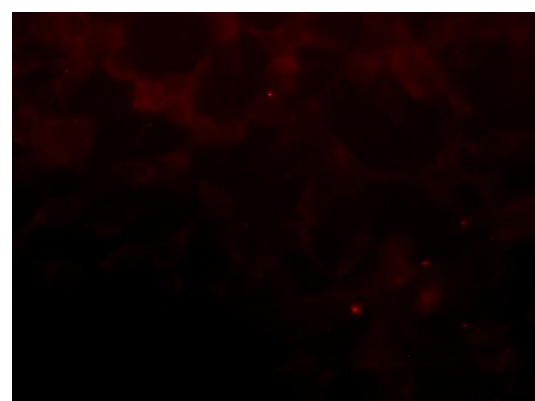

DAPI

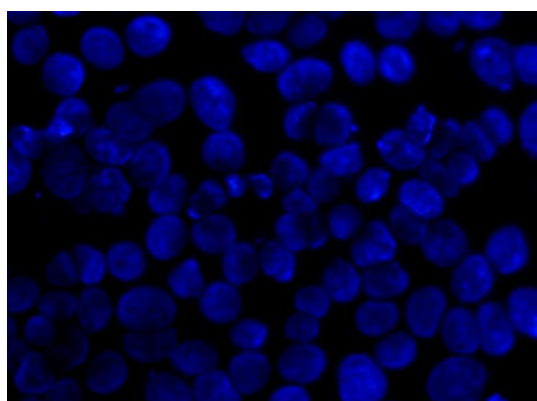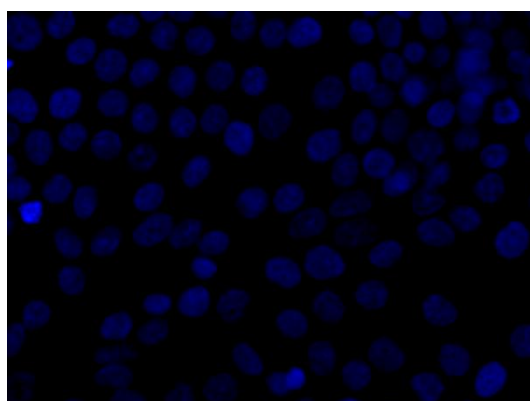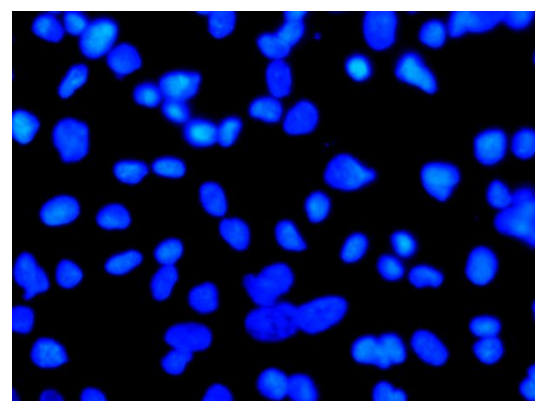

merge

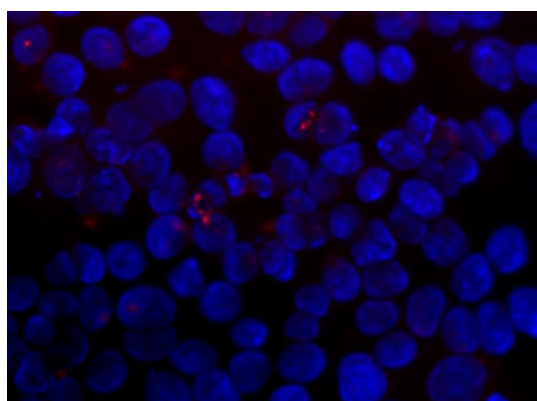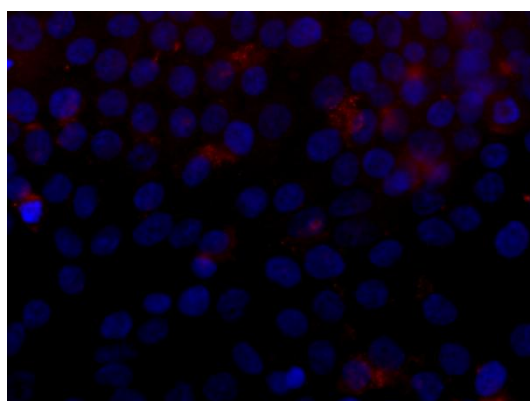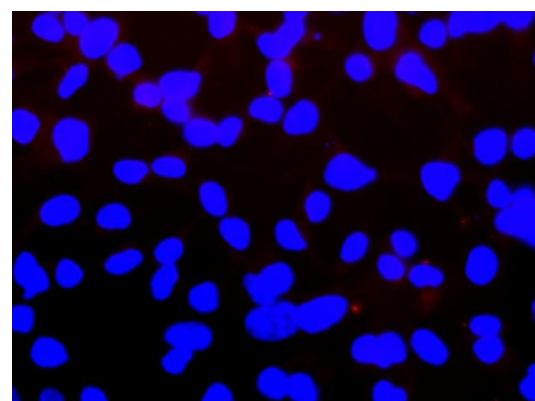

Supplement: Supplementary file 1 — Additional file 1: Figure S1: Immunocytochemical staining of ANKH and PANX1, nuclei are stained with DAPI. Representative images are shown, the bar represents 100 μm. (PDF 447 KB) [file 12943_2014_1463_MOESM1_ESM.pdf]

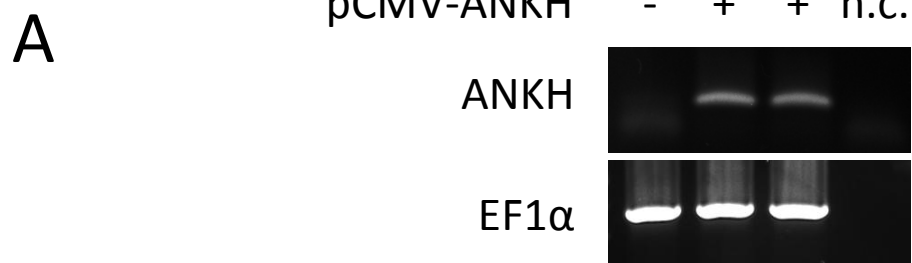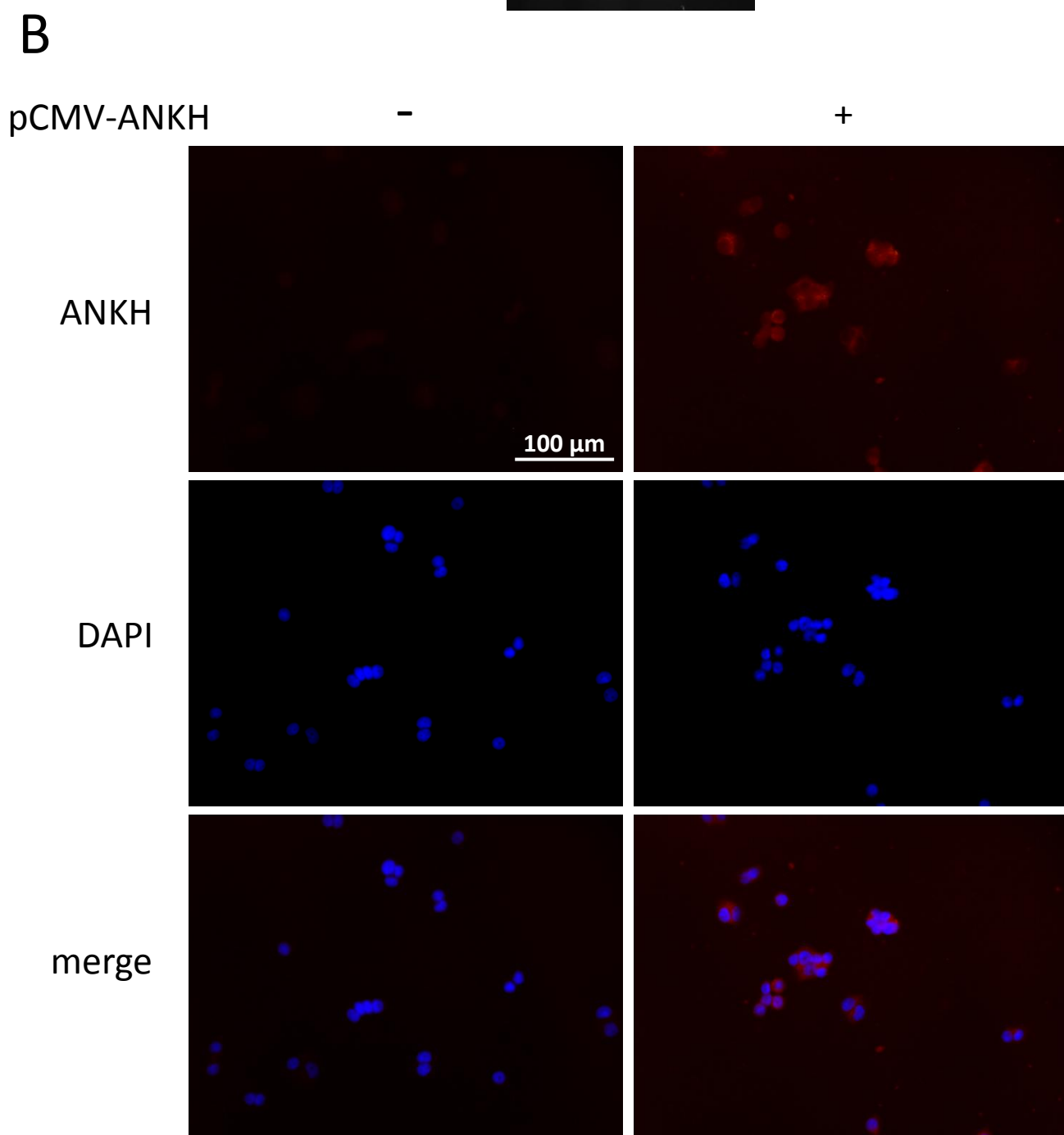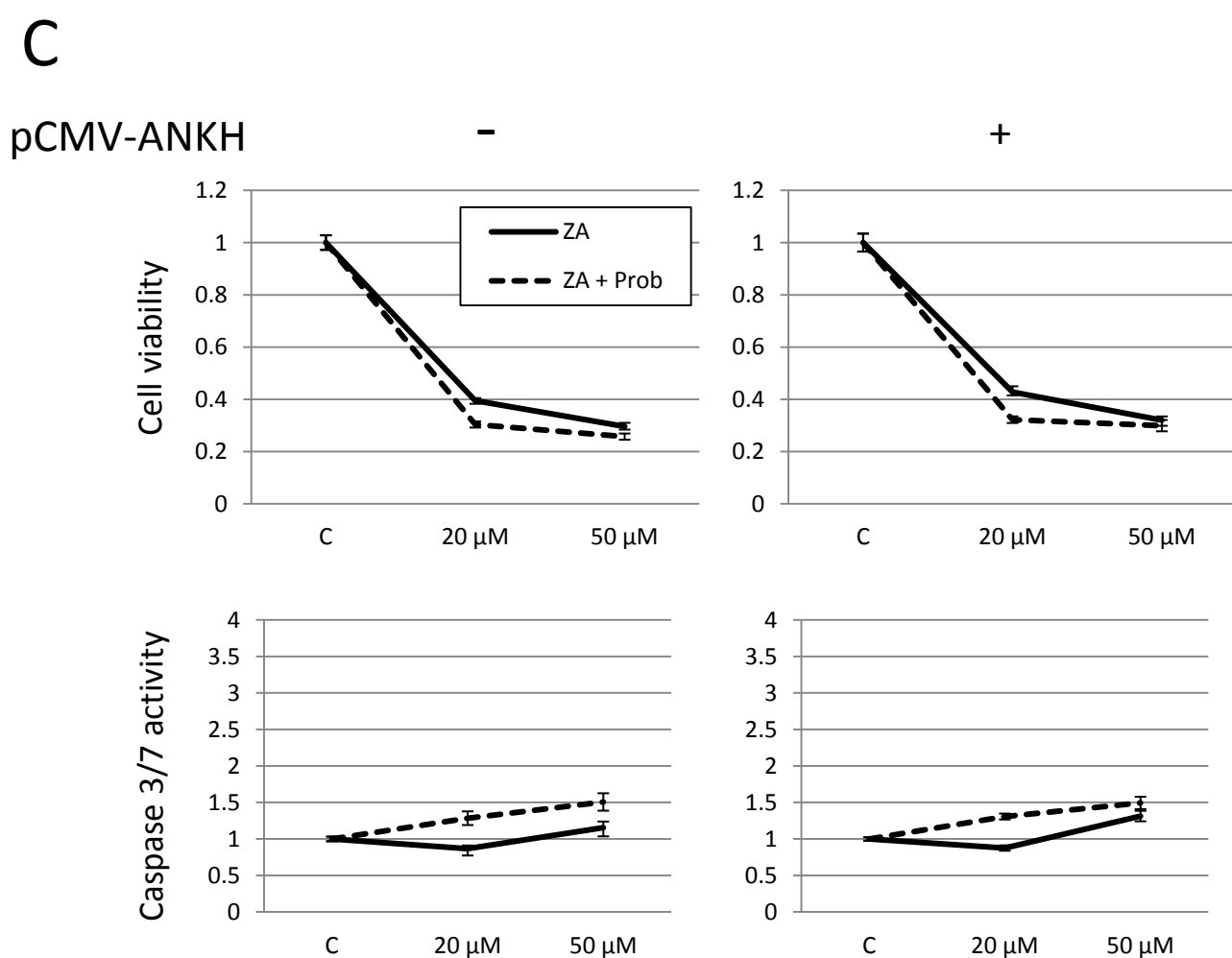

Supplement: Supplementary file 2 — Additional file 2: Figure S2: Overexpression of ANKH in T47D cells. A) Amplification of ANKH in pCMV-ANKH and pCMV stable cell lines. EF1α was used as a housekeeping gene. B) Immunocytochemical staining of ANKH in pCMV-ANKH and pCMV stable T47D cells. Nuclei are stained with DAPI. Representative images are shown, the bar represents 100 μm. C) Cell viability and caspase 3/7 activity in pCMV-ANKH and pCMV stable T47D cells co-treated with probenecid and ZA. All data are expressed as means of six different measure points of three independent experiments as percent of controls ± SEM. BP: bisphosphonate, black line; Prob: probenecid, dotted line probenecid co-treatment. (PDF 344 KB) [file 12943_2014_1463_MOESM2_ESM.pdf]
